# Whole-Genome Assemblies of 16 Burkholderia pseudomallei Isolates from Rivers in Laos

**DOI:** 10.1128/MRA.01226-20

**Published:** 2021-01-28

**Authors:** Nicole Liechti, Rosalie E. Zimmermann, Jakob Zopfi, Matthew T. Robinson, Alain Pierret, Olivier Ribolzi, Sayaphet Rattanavong, Viengmon Davong, Paul N. Newton, Matthias Wittwer, David A. B. Dance

**Affiliations:** a Spiez Laboratory, Swiss Federal Office for Civil Protection, Spiez, Switzerland; b Interfaculty Bioinformatics Unit, Department of Biology, University of Bern, Bern, Switzerland; c Lao-Oxford-Mahosot Hospital-Wellcome Trust Research Unit, Microbiology Laboratory, Mahosot Hospital, Vientiane, Laos; d Department of Environmental Sciences, Biogeochemistry, University of Basel, Basel, Switzerland; e Department of Medical Microbiology, Amsterdam University Medical Centers, Amsterdam, The Netherlands; f Centre for Tropical Medicine and Global Health, Nuffield Department of Medicine, University of Oxford, Oxford, United Kingdom; g Faculty of Infectious and Tropical Diseases, London School of Hygiene and Tropical Medicine, London, United Kingdom; h iEES-Paris (IRD, Sorbonne Universités, UPMC Univ Paris 06, CNRS, INRA, UPEC, c/o Department of Agricultural Land Management (DALaM), Vientiane, Laos; i Géosciences Environnement Toulouse (GET), Université de Toulouse, IRD, CNRS, UPS, Toulouse, France; University of Maryland School of Medicine

## Abstract

We report 16 Burkholderia pseudomallei genomes, including 5 new multilocus sequence types, isolated from rivers in Laos. The environmental bacterium B. pseudomallei causes melioidosis, a serious infectious disease in tropical and subtropical regions. The isolates are geographically clustered in one clade from around Vientiane, Laos, and one clade from further south.

## ANNOUNCEMENT

Burkholderia pseudomallei causes the human infectious disease melioidosis and is found in tropical and subtropical soils and freshwater (1). Survival and replication in various ecological niches and within hosts is possibly enabled by the large and highly variable accessory genome of B. pseudomallei (2, 3). Genome descriptions of B. pseudomallei isolates contribute to research on links between environment-associated and disease-associated genes of B. pseudomallei and their functions (3, 4).

**FIG 1:**
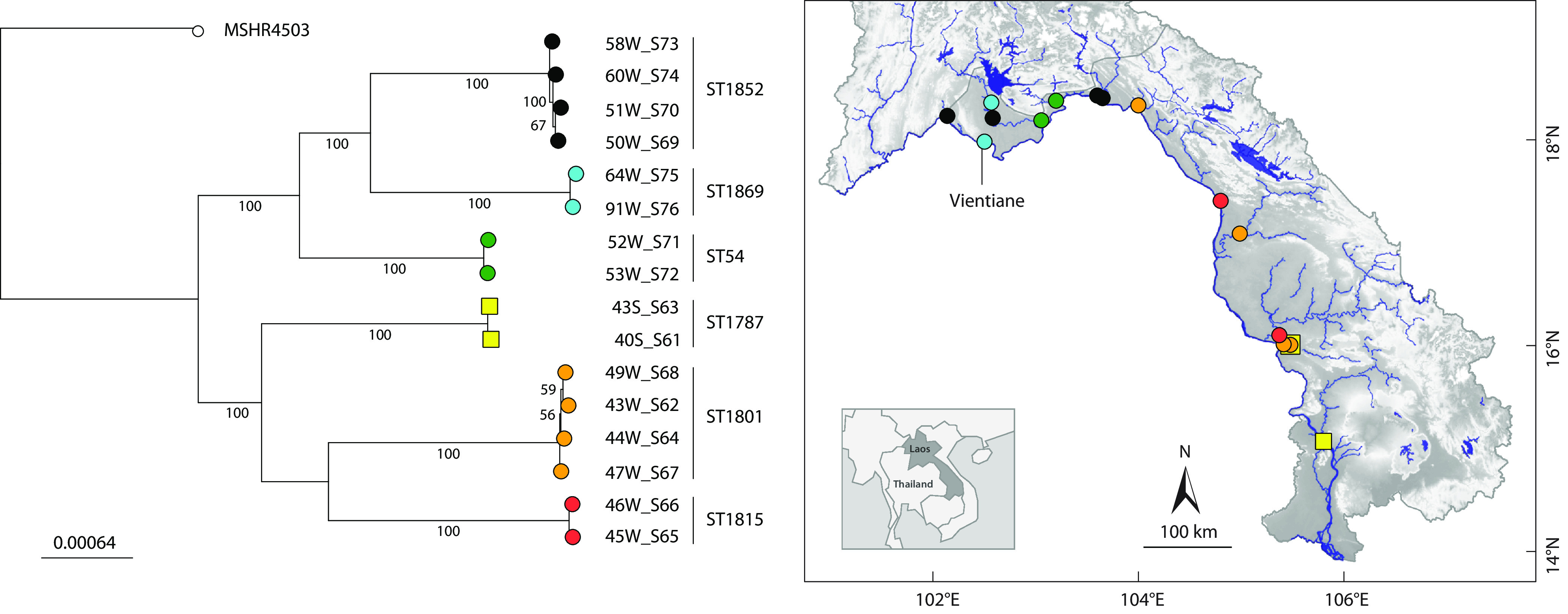
Maximum likelihood phylogeny and geographic locations of 16 environmental B. pseudomallei isolates. Clusters of isolates are displayed in different colors and correspond to sequence types (ST); circles represent water isolates, and squares represent sediment isolates. B. pseudomallei strain MSHR4503 from northern Australia (15) was used as the reference. The tree scale indicates changes per nucleotide; bootstrap values were calculated from 1,000 bootstrap replicates and are reported as percentages. The phylogenetic tree was visualized using Microreact (17), and the map was adapted from reference 5.

We sequenced the genomes of 16 B. pseudomallei isolates from 14 filtered water samples and two sediment samples from rivers in Laos, cultured and confirmed as previously described (5). After storage at −80°C and pure culture on nutrient agar in air at 37°C for 24 h, genomic DNA was extracted using the Qiagen DNeasy blood and tissue kit and submitted to Microsynth AG (Balgach, Switzerland) for Nextera XT library preparation and sequencing using an Illumina NextSeq 500 instrument (paired-end [PE], 150-bp reads). Reads were quality trimmed using Trimmomatic 0.36 (slidingwindow:4: 8, minlen:127) (6) and assembled using SPAdes 3.11.1 (-careful, -mismatch-correction, -k 21, 33, 55, 77, 99, 127 bp) (7). Pilon 1.22 (8) was applied to improve the quality of the draft assemblies. Scaffolds of <200 bp or with low coverage were removed. Finally, contaminants were removed manually using a BLAST search against the NCBI nucleotide database. The quality and completeness of the *de novo*-assembled genomes were accessed using BUSCO 3.0.1 (lineage, *Betaprotebacteria* odb9) (9), and basic assembly statistics were compared using QUAST 4.6.3 (10). The genomes were annotated automatically using the NCBI Prokaryotic Annotation Pipeline 4.11 (11). Default settings were used for all software unless otherwise specified. A summary of the assembly results is provided in Table 1. The 16 isolates were found to belong to 6 different sequence types using the multilocus sequence typing pipeline (12, 13), 5 of which were new. Sequence type 54 (ST54) (two isolates) was previously described and is common in neighboring Thailand (14). To unravel the phylogenetic relationship of the isolates, we first constructed a core single nucleotide polymorphism genome alignment using Snippy 4.4.3 with B. pseudomallei MSHR4503 (15) as the reference. Then, we built a maximum likelihood tree using RAxML 8.2.11 (16) with a general time-reversible nucleotide substitution model including 1,000 bootstraps (Fig. 1). The six main branches of the tree correspond to the sequence types and are geographically clustered in two different clades. One clade includes isolates from or around Vientiane, Laos (city and province), whereas the other consists of isolates from further south. The sediment isolate from Xe Bangnouan, Laos, is more closely related to the sediment isolate from the Mekong River than to the corresponding water isolate (Fig. 1). However, with relatively few samples taken at one point in time from rivers with large catchment areas, the interpretation of these clusters remains speculative. It is hoped that sequencing more isolates of B. pseudomallei from Laos will improve our understanding of the phylogeography of the organism within the country and enable comparisons to be made between clinical and environmental isolates.

**TABLE 1:** Characteristics and accession numbers of the genomes of 16 B. pseudomallei isolates from rivers in Laos

Isolate^*a*^	River^*a*^	Latitude^*a*^	Longitude^*a*^	Raw read SRA no.	GenBank assembly accession no.	Sequence type	No. of contigs	Assembly length (Mbp)	GC content (%)	*N*_50_ (kbp)	No. of paired reads (million)	Genome coverage (×)
40S_S61	Mekong	15.11316	105.80506	SRR11097786	GCA_014713055.1	ST1787	186	7.15	68.19	119.6	4.8	203
43W_S62	Xe Bangnouan	16.00286	105.47903	SRR11097785	GCA_014713065.1	ST1801	205	7.17	68.14	107.1	4.7	183
43S_S63	Xe Bangnouan	16.00286	105.47903	SRR11097778	GCA_014713085.1	ST1787	176	7.15	68.19	128.2	4.4	195
44W_S64	Mekong	16.00421	105.42515	SRR11097777	GCA_014713025.1	ST1801	191	7.17	68.14	123.3	5.5	231
45W_S65	Xe Banghieng	16.09798	105.37699	SRR11097776	GCA_014713015.1	ST1815	184	7.15	68.15	126.5	4.6	194
46W_S66	Mekong	17.39898	104.80098	SRR11097774	GCA_014712945.1	ST1815	188	7.15	68.14	132.9	5.3	219
47W_S67	Xe Bangfai	17.07787	104.98503	SRR11097775	GCA_014712955.1	ST1801	209	7.17	68.15	119.7	5.3	221
49W_S68	Nam Kading	18.32559	104.00002	SRR11097773	GCA_014712965.1	ST1801	193	7.17	68.14	127.8	5.3	223
50W_S69	Nam Xan	18.39103	103.65572	SRR11097772	GCA_014712935.1	ST1852	192	7.24	68.1	138.5	5.0	208
51W_S70	Nam Gniep	18.41756	103.60212	SRR11097771	GCA_014712915.1	ST1852	222	7.25	68.26	110.8	4.9	201
52W_S71	Nam Mang	18.37017	103.19838	SRR11097784	GCA_014712835.1	ST54	168	7.04	68.09	134.4	5	213
53W_S72	Nam Ngum	18.17874	103.05594	SRR11097783	GCA_014712895.1	ST54	182	7.04	68.27	126.7	4.4	188
58W_S73	Nam Ngum	18.20194	102.58669	SRR11097782	GCA_014712875.1	ST1852	184	7.25	68.1	120.9	4.7	195
60W_S74	Nam Sang	18.22297	102.14228	SRR11097781	GCA_014712825.1	ST1852	186	7.24	68.11	123	4.6	189
64W_S75	Mekong	17.97309	102.50404	SRR11097780	GCA_014712815.1	ST1869	212	7.3	68.1	97.6	4.8	197
91W_S76	Nam Ngum	18.3555	102.57198	SRR11097779	GCA_014712775.1	ST1869	194	7.21	68.11	87.9	4.1	171

a Data from reference 5.

### Data availability.

Illumina raw reads and genome assemblies were deposited at the NCBI and DDBJ/ENA/GenBank, respectively. The accession numbers are listed in Table 1. The isolates are linked to the respective sequence types on the PubMLST database.
